# Features of mania in comorbidity of bipolar disorder and borderline disorder

**DOI:** 10.1192/j.eurpsy.2026.11187

**Published:** 2026-07-31

**Authors:** S. Sorokin, A. Churkina, M. Popov

**Affiliations:** Department for the Study of Endogenous Mental Disorders and Affective States, Mental health research center, Moscow, Russian Federation

## Abstract

**Introduction:**

Bipolar disorder is characterized by a high frequency of comorbidity with other mental illnesses, in particular, with borderline disorder. In clinical practice, the condition of every fifth patient with bipolar II disorder and every tenth patient with bipolar I disorder also meets the diagnostic criteria for borderline disorder. Comorbidity of bipolar disorder with personality disorder leads to decreased effectiveness of therapy, patient incompliance, increased risk of alcohol abuse and suicidal behavior. Manic states are particularly difficult in people with emotionally unstable traits, which include their inherent impulsiveness, abnormal behavior, irritability, and conflict.

**Objectives:**

To determine the characteristics of manic states in the structure of bipolar disorder in individuals with borderline features.

**Methods:**

The sample consisted of, 30 patients (22 women, 8 men) who were on inpatient or outpatient treatment at the clinic since 2022 to 2024. The examination of patients was carried out using clinical-psychopathological, clinical-anamnestic methods in connection with the presence of diagnosed bipolar and borderline disorders.

**Results:**

Among the patients we examined, all had previously experienced manic phases, according to their medical history. Only in 7 patients included in the study, the current hospitalization was due to the presence of signs of a manic state, in 14 examined patients the reason for hospitalization was an affective mixed state, and in 9 the current phase corresponded to the criteria for depression. Manifestations of borderline disorder were observed in all subjects included in this study, regardless of affect. All patients in the manic phase showed signs of behavioral disturbances associated with increased drives (substance abuse, promiscuity, kleptomania). Patients showed pronounced distractibility, emotional insensitivity, and empathy deficiency. There was also a tendency to downplay the severity of the disease manifestations, focusing on the subjectively experienced side effects of the previously conducted therapy, and the desire to evoke sympathy. In the manic state before the start of drug treatment, persistent disruption of night sleep, decreased appetite with fairly rapid weight loss or, conversely, overeating without fear of weight gain and other consequences were observed.

**Conclusions:**

Manic states in individuals with borderline features were characterized by quite pronounced behavioral disorders, a distinct dysphoric component, eating disorders, and the formation of pathological dependencies. It follows that comorbidity can impart “pseudo” mixed features to a manic state, which creates additional diagnostic, therapeutic, and prognostic difficulties.

**Disclosure of Interest:**

None Declared

